# Magnetic resonance imaging applications to amyotrophic lateral sclerosis beyond the central nervous system

**DOI:** 10.3389/fneur.2026.1849783

**Published:** 2026-08-25

**Authors:** Jan Kassubek, Hans-Peter Müller

**Affiliations:** 1 Department of Neurology, University Hospital Ulm, Ulm, Germany; 2 Department of Nuclear Medicine, University Hospital Ulm, Ulm, Germany; 3 Department of Diagnostic and Interventional Radiology, University Hospital Ulm, Ulm, Germany

**Keywords:** body composition, body fat, magnetic resonance imaging, motor neuron disease, muscle, muscle imaging, tongue

## Abstract

Traditionally, in amyotrophic lateral sclerosis (ALS) and other motor neuron disorders, the diagnostic and research focus in the domain of magnetic resonance imaging (MRI) has been targeted on the brain and spinal cord. Nevertheless, structures outside the central nervous system (CNS) are core structures of the pathological processes and are also assessible by imaging approaches. Advanced imaging of structures like bodyfat and muscles can considerably contribute to the pathophysiological understanding and disease monitoring of ALS. The multiparametric combination of MRI approaches that capture these changes could be very helpful in future studies, especially when machine learning algorithms are used. This review summarizes the current state of the art and the role of imaging beyond the CNS in ALS, examining its diagnostic and monitoring potential and how it might ultimately lead to improved management strategies for patients.

## Background

**Figure 1:**
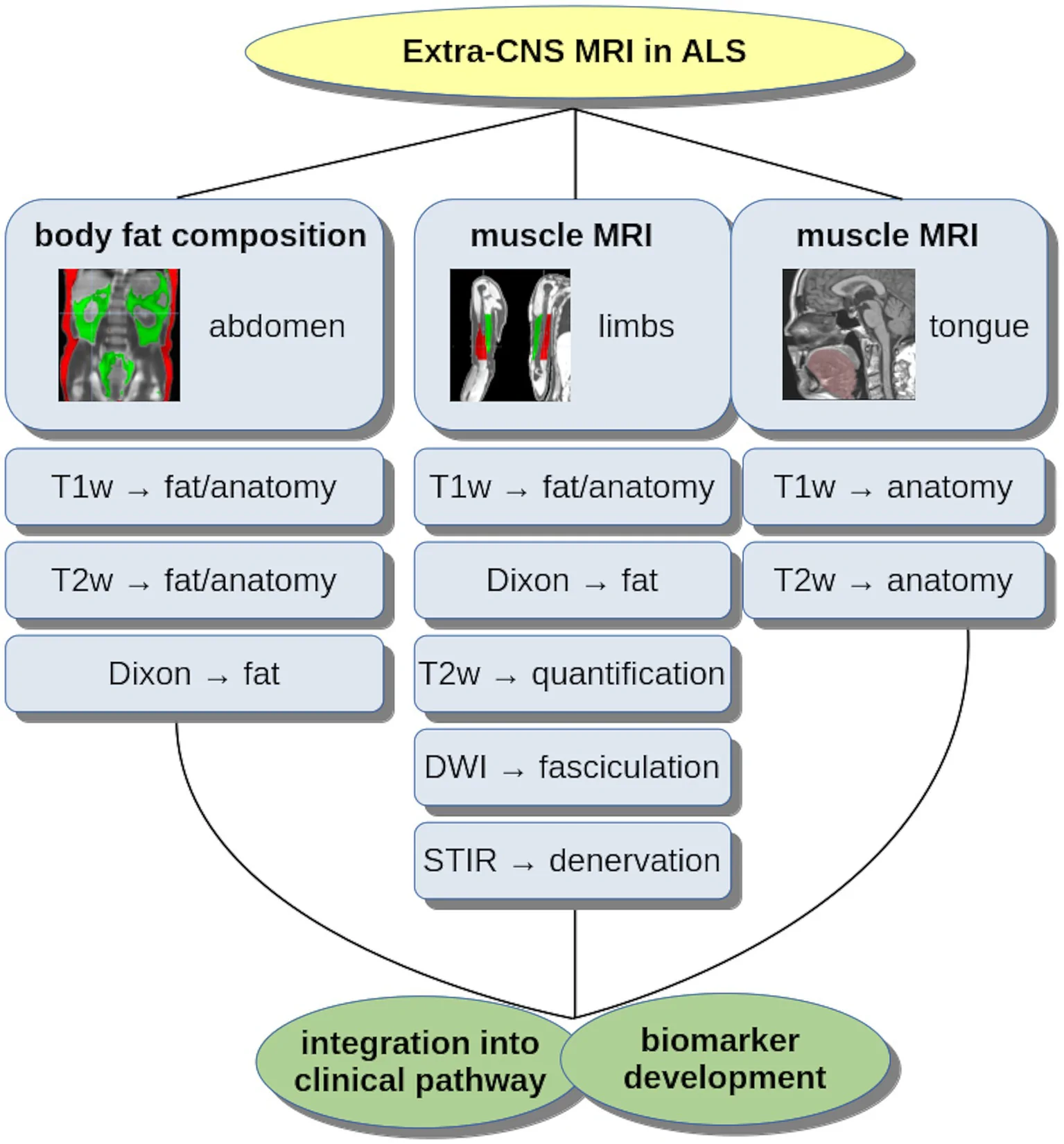
The role of extra-CNS imaging in ALS. MRI of body fat compartments and muscle MRI including limb and tongue MRI, respectively, are promising techniques to be integrated into clinical pathway and for biomarker development. CNS, central nervous system; MRI, magnetic resonance imaging; ALS, amyotrophic lateral sclerosis; DWI, diffusion weighted imaging; STIR, Short Tau Inversion Recovery.

Amyotrophic lateral sclerosis (ALS) is a fatal neurodegenerative disorder with an incidence of up to 3 per 100,000 per year which predominantly affects individuals aged 60–75, with sporadic and genetic variants and different clinical phenotypes (1). In ALS and other motor neuron disorders (MND), traditionally, the diagnostic and research focus in the domain of imaging has been targeted on the central nervous system (CNS), i.e., the brain and the spinal cord. There has been considerable clinical and academic progress in that field over the recent years, addressing longitudinal propagation patterns (with specific factors of genotype), pathological staging, correlates of clinical phenotype and disease burden, adaptive changes and reserve, as recently reviewed (2, 3). Current directions also include early brain and spinal cord changes in presymptomatic mutation carriers (4). However, beyond the neuroanatomical structures addressed by the vast majority of these advanced imaging studies, magnetic resonance imaging (MRI) of structures outside the CNS like muscles and bodyfat can considerably contribute to the efforts to further understand ALS pathology and finally to improve clinical management. The combined diagnostic assessment of different parameters is essential to capture the various aspects of a multidimensional disease like ALS. The concept of *extra-CNS* imaging in MND is reviewed in the following, addressing its applications, challenges, and potential future directions (Figure 1).

## Neuroimaging applications to ALS beyond the CNS

### Bodyfat composition – clinical observations

Malnutrition and weight loss are frequent in ALS and strongly predict accelerated progression and earlier mortality. ALS patients often exhibit altered resting energy expenditure which is coupled with dysphagia and reduced intake, leading to weight and fat loss (5, 6). Body mass index (BMI)-losing phenotypes displayed dysregulation in lipid pathways and lipoprotein profiles (e.g., LDL-C, apolipoprotein B), also correlated with ALS risk and survival (7). Weight loss at diagnosis, which also occurs independent of dysphagia, is a multifactorial, individual process which has been shown to be related to survival in several studies (8). It has been demonstrated that the body weight starts to decrease already about ten years before the first motor symptoms (9) and that, on the other hand, high body mass index is a positive prognostic factor in ALS (10). These findings are also evident in animal models of ALS (11); in the TDP-43^A315T^ mouse model, significant histological alterations in adipose tissue prior to the symptomatic stage of the disease have been demonstrated (12). These conceptual approaches have led to therapeutic interventions, e.g., to investigate the efficacy of a high-caloric fatty diet for increasing survival in ALS which demonstrated, although no evidence for a life-prolonging effect was shown for the whole study population, a significant survival benefit for the subgroup of fast-progressing patients (13), further corroborated by the reversed course of NfL serum levels for high-caloric fatty diet and placebo measured during the intervention (14).

While early research in the field of muscle MRI primarily provided qualitative findings, the development of advanced MRI methods has increasingly led to the use of quantitative methods. The MRI Short Tau Inversion Recovery (STIR) signal intensity was shown to be sensitive enough for detection of topography of the nerve lesion (15, 16). Diffusion weighted sequences allow for time-resolved recording of spontaneous muscular contractions and provide new insights into their dynamic course and fasciculation (17, 18).

Adipose tissue is both an energy store (white adipose tissue) and has specialized functions in thermogenesis (brown adipose tissue), and it is an endocrine organ which secretes a variety of soluble mediators, with a critical role in the physiological regulation of the metabolism acting on the hypothalamus (19). In that context, it is most intriguing that it has been demonstrated by several groups in the last decade that the hypothalamus, as a core CNS structure modulating appetite and metabolism, is involved in the neuropathological process of ALS and is significantly atrophied in ALS patients (20–23). MRI-based quantitative analysis of the hypothalamic volume shows that hypothalamic atrophy is related to BMI and age at onset in ALS; decreased anterior hypothalamic volume is associated with earlier onset of disease (21). Reduced hypothalamic volume correlates with lower BMI, suggesting unique neurobiological underpinnings of fat loss in ALS (20). In addition to hypothalamic MRI studies, a recent multiparametric neuroimaging study (of the CNS) has addressed functional changes of the neurophysiology of food intake processing/appetite in ALS (24).

### Body fat composition – imaging access to body fat compartments

BMI is a general measure which lacks sensitivity in distinguishing fat vs. lean tissue. Imaging of the bodyfat compartments has been increasingly used in ALS for body composition analysis, in addition to anthropometric measurements which are used to assess nutritional status (25). Tools like computed tomography (CT) and dual-energy X-ray absorptiometry (DEXA) revealed that quantitative and qualitative adipose changes (especially subcutaneous fat loss or adipopenia) are predictors of progression and survival: in ALS, imaging studies by use of DEXA identified that both increments and decrements in fat mass and body fat percentage correlated with disease progression; in particular, declining fat mass was associated with more rapid progression (26). Since the influence of bodyfat on survival in ALS is not only depending on the total amount of body fat but significant evidence has been found that the body fat composition, i.e., the discrimination of visceral and subcutaneous fat compartments, could be essential on the effects of survival in ALS, an MRI study targeted both compartments separately (27). For this study, an operator-independent and reproducible automatic MRI analysis technique was used which had been developed to separately determine the subcutaneous and visceral fat volumes in selected body regions for whole-body-based MRI (28) and was used in several bodyfat analyses in various conditions since then (e.g., (29)). In the application to 62 ALS patients, it could be demonstrated that the ALS patients had more visceral and less subcutaneous fat and importantly, that higher subcutaneous fat correlated with better ALSFRS-R scores and predicted longer survival (an effect which was significant in males only, however) (27). The reason why these changes were observed only in males could not be dissolved by this imaging-based study and might be biased by the limited sample size. A current study using bioelectrical impedance analysis in 93 ALS individiduals (29 females) reported a lower association between fat mass and ALSFRS-R in males compared to females (30), but further study is necessary. To date, although gender differences in body composition and metabolic responses are acknowledged, no gender specific findings exist in studies which assess body composition changes in ALS, including presymptomatic gene carriers (31). These findings suggest that some fat stores play a protective role in ALS pathogenesis and are in support of potential benefits of dietary interventions. A recent study used a deep learning-based volumetric CT body composition analysis to determine the fat volume index and skeletal muscle index for the abdominal area in 80 ALS patients (32). Here, adipopenia was observed which was associated with shorter survival and was thus confirmed to be an independent poor prognostic factor for survival. By integrating body composition analysis in future multimodal approaches (by MRI, but also by DEXA or CT) with functional scales, clinicians will probably better identify those at risk, tailor macronutrient plans, and monitor therapy (e.g., via bioelectrical impedance).

### Muscle involvement – clinical observations

The value of muscle MRI has been extensively explored in the diagnostic procedures of myopathies (33–35). In MND including ALS, the degeneration of motor neurons results in muscle denervation with associated muscle changes structurally visible as edema, fatty infiltration, fibrosis, and atrophy, and muscle MRI appears to be a promising tool to study muscle pathophysiology (36). In ALS, muscle involvement is not merely a consequence of motor neuron degeneration but also reflects intrinsic muscular changes. Early in the disease, muscles exhibit denervation and reinnervation processes, leading to alterations in muscle composition and function. The concept of muscle imaging in ALS refers to the utilization of imaging to assess muscle integrity and to identify early signs of disease (37). In that context, T1-weighted muscle MRI has already been applied to distinguish ALS/MND patients from controls (37, 38). In ALS, the muscles undergo a complex process of denervation, reinnervation, and eventual degeneration which may not be immediately apparent through routine clinical assessment alone. As a result, the advent of advanced imaging techniques has paved the way for earlier detection, more precise monitoring of disease progression, and better differentiation from other neuromuscular disorders (34, 37, 38). Already in early disease stages, whole-body muscle MRI with T2-weighted and diffusion-weighted imaging showed signal hyperintensities, mainly in the upper and lower limbs (39) so that it has been concluded that this approach might facilitate early diagnosis and be used for pre-EMG mapping in order to choose suitable target muscles. A recent review (36) summarized that muscle MRI provides a tool to study muscle pathophysiology in MND, but further research is required to define its place in clinical and research practice. It is important to note that other MND like spinobulbar muscular atrophy (SBMA) or spinal muscular atrophy (SMA) have shown selective muscle damage patterns in MRI as a potential option to facilitate the diagnostic procedures, for example by motor unit magnetic resonance imaging (MUMRI) (40). In SBMA, quantitative 3-point Dixon and semiquantitative T1-weighted and short tau inversion recovery imaging demonstrated significant fat infiltration in bulbar and lower limb muscles as a characteristic pattern of muscle involvement, different from ALS (16). To explore the reasons for these differences in muscle fat infiltration between ALS and SBMA, further studies are needed - the disease progression which in general in ALS is much quicker than in SBMA can be presumed as an important contributing factor. In SMA, muscle MRI has demonstrated a combination of muscle atrophy and muscle infiltration with with atrophy patterns characterized by a various degree of proximal to distal gradient (41). For such a differentiation between different MND, in addition to mapping the damage in a given muscle or group of muscles alone, the patterns of muscle involvement can be identified by MRI-based approaches. Beyond this differentiation of MND, this concept is also a valuable tool to standardize and quantify the differential involvement or propagation scheme of the musculature, in addition to clinical assessment. As one straightforward approach, the cross-sectional area of the cervical muscles was measured on axial T1-weighted MRI at the C3 vertebra level as a so-called cervical Skeletal Muscle Index and demonstrated lower values in ALS with a disease duration of more than six months after symptom onset (42). These results add to the concept of MRI-based assessments of the cervical muscles which has been developed for dropped head syndrome in association with various conditions including cervical spine damage (43). The MRI-based analysis of ALS-associated regional muscle changes has been investigated for temporal muscle thickness calculated on T1-weighted MRI which was significantly lower in ALS patients than in healthy subjects and which was considered to be a surrogate biomarker of survival and functional status in ALS (44). Beyond such a region-wise analysis to assess the condition of the muscles, the motor deficits/pareses in the course of ALS are conceptually considered to follow specific patterns. This indicates that ALS might primarily involve those muscle groups that receive the strongest direct corticomotoneuronal innervation via corticofugal tracts. Thus, ALS as a network disorder preferentially affects corticospinal projections providing direct monosynaptic input to *α*-motoneurons, as investigated in a clinical study (45). In order to analyze this clinical pattern of muscle involvement and its potential association with the neuroanatomical staging of corticofugal involvement in the disease, a whole-body muscle MRI study was performed which assessed different parameters (i.e., volume, fat fraction, and functional remaining muscle area) in muscle groups with different corticomotoneuronal input (46). Indeed, the muscles receiving stronger direct corticomotoneuronal input showed more pronounced morphological involvement than the muscles with less monosynaptic corticomotoneuronal input (best discriminative parameter, functional remaining muscle area (fRMA)). Thus, whole-body MRI-based muscle analysis, i.e., parameterization and quantification of muscle alterations from MRI, provided additional evidence for a characteristic pattern of pareses in ALS in support of the future aim that muscle MRI might be developed as a biomarker for ALS (46). It is of note that in this study, only a small subset of ALS individuals (3/33) had a bulbar onset so that the question is still open how bulbar patients might differ when compared to those with limb onset. Despite these promising results, muscle MRI in ALS has to be investigated in longitudinal design in order to assess the utility and responsiveness of approaches like quantitative muscle MRI in order to potentially qualify as an outcome measure in ALS clinical trials. In that respect, Diaz and colleagues used 3-point Dixon fat-water and T2 relaxometry 3 *T* MRI data to perform a longitudinal quantitative muscle mapping in ALS patients (47). Their study demonstrated that the MRI-based outcome measures had a higher responsiveness at 6 months than the clinical score ALS functional rating scale (revised) (ALSFRS-R) as based on absolute standardized response means values so that the authors concluded that quantitative muscle MRI has the potential to be used as complimentary outcome measure in 6-month clinical trials in ALS. However, given the limited sample size (n = 17 with only 11 patients completing three serial scans), this perspective has to await further longitudinal MRI studies. In addition to muscle MRI alone, peripheral nerve imaging by magnetic imaging neurography has been established and has become a favorable method to assess peripheral nerve inflammation and degeneration. By this approach (using diffusion-weighted and high-resolution fat-saturated T2-weighted and multi-echo T2-relaxometry sequences), multiple peripheral nerves of the upper and lower limbs were investigated in patients with ALS and compared with patients with multifocal motor neuropathy (MMN), demonstrating that MMN patients had nerve enlargements while there were no nerve alterations in ALS (48). As a combination of the approaches of both nerve (plexus) and muscle MRI, by quantitative MRI of the brachial plexus and limb-girdle muscles in the affected limbs of patients with ALS, the cross-sectional areas of the brachial plexus roots, trunks, and cords and the nerve-muscle T2 signal intensity ratio of the brachial plexus trunks were significantly smaller than in controls and the muscles showed significantly smaller cross-sectional areas and higher fat fraction (49, 50). As an important application of diffusion MRI to MND, multiple-point diffusion-weighted stimulated echo imaging has been developed to measure spontaneous mechanical activities and fasciculations (51). A novel approach in ALS is motor unit MRI/MUMRI as a non-invasive technique to detect fasciculations which uses a pulsed gradient spin-echo diffusion weighted sequence in which motor unit contractions manifest as transient signal voids within skeletal muscles; this technique detected a more than 10-fold elevation in fasciculation rate in the biceps brachii, lumber paraspinal muscles, and lower leg muscles of 10 ALS patients compared to controls (52).

Advanced MRI techniques can measure skeletal muscle inflammation/necrosis and fat infiltration (which are strong indicators of disease activity and progression in many neuromuscular disorders) by muscle T2 relaxometry and water-fat separation techniques, respectively. Differences between water and fat T1 and T2 relaxivities were assessed by applying a bi-component extended phase graph fitting approach to simultaneously quantify the muscle water T2 and fat fraction from standard multi-slice multi-echo acquisitions in the presence of stimulated echoes (53).

In summary, muscle MRI can help to visualize changes in the muscles, such as muscle atrophy or changes in muscle tissue, in order to define and quantify patterns of involvement in order to better understand the progression of the disease. However, the prognosis for ALS is very individual and depends on many factors, such as the phenotype, the age of the patient, and other medical aspects (like body composition). Therefore, muscle MRI tends to be used as a supplement to obtain a comprehensive picture, but not primarily for prognosis yet.

### Specific muscle involvement – tongue involvement

A complex muscular organ is the tongue, essential for speech and swallowing, and given that is controlled by cranial nerve XII it is specifically involved in the pathological process of MND. Bulbar dysfunction, characterized by tongue wasting and fasciculation, accompanied by flaccid dysarthria and dysphagia, is emerging in the vast majority of patients during the advanced phases of ALS (54). Beyond its occurrence in advanced `classical` ALS (1), bulbar involvement might be even prominent in ALS variants like progressive bulbar palsy (PBP) in which patients show an isolated bulbar onset with a progressive affection of the lower cranial nerves including tongue atrophy (55) before they develop spinal MND symptoms. Prognostic biomarkers of bulbar involvement are needed, since it is one of the key determinants of long-term prognosis and survival in this disorder (56).

As early as 1989, tongue changes in shape and T1 signal were described in MRI investigations in ALS patients (57). Later, there were casuistic MRI findings reported as “bright tongue sign,” i.e., a diffuse T1-weighted hyperintensity of the tongue indicating fatty infiltration of tongue muscles in ALS (58, 59). Although it has been shown by ultrasound that the tongue thickness in ALS is reduced compared to controls (60), tongue size and shape can significantly vary across subjects. An MRI-based imaging dataset for investigations of the tongue musculature, including multi-contrast and multi-site MRI data from controls, has been retrospectively collected to improve the possibility of investigating the tongue role in physiological processes and speech production (61). Some systematic MRI studies have suggested structural tongue measures in the course of ALS (62). In a large study in 175 ALS patients, specifically, T1 sequences were used to assess atrophy, fibrosis and fatty degeneration, and a previous large-scale study suggested that *in vivo* sonography and region-of-interest (ROI)-based MRI tongue measures could aid as biomarkers to reflect bulbar and motor function impairment in ALS (63). Recently, Shaw and colleagues used multi-contrast MRI for the measurement of the volumes of key tongue muscles in patients with MND by the application of a novel segmentation and normalisation method for the tongue using standard T1-weighted or T2-weighted 3 *T* MRI data from a multi-site dataset (61, 64). By this technique, the reliable segmentation of five large muscles within the tongue was possible, resulting in the demonstration of gross volumetric evidence for tongue degeneration in MND subtypes. A recent study in the PBP phenotype of ALS showed significantly atrophied tongue volumes by a Convolutional Neural Network (CNN)-based automated MRI tongue volume quantification (65). On the other hand, motor unit MRI could not demonstrate significant abnormalities in the tongues of MND patients (52). In other MND like SBMA, significant atrophy of the tongue was observed; this atrophy correlated with total SBMA-functional rating scale and even more with bulbar subscores (66).

In summary, as a specific muscle imaging approach, MRI-based tongue volume determination might be used as a disease severity marker in future longitudinal clinical studies. To this end, larger patient groups have to be investigated, and the structure/volume of the tongue will have to be correlated with tongue functional assessments including tongue movement ability and swallowing function (65).

### Technical considerations: MRI

**Figure 2:**
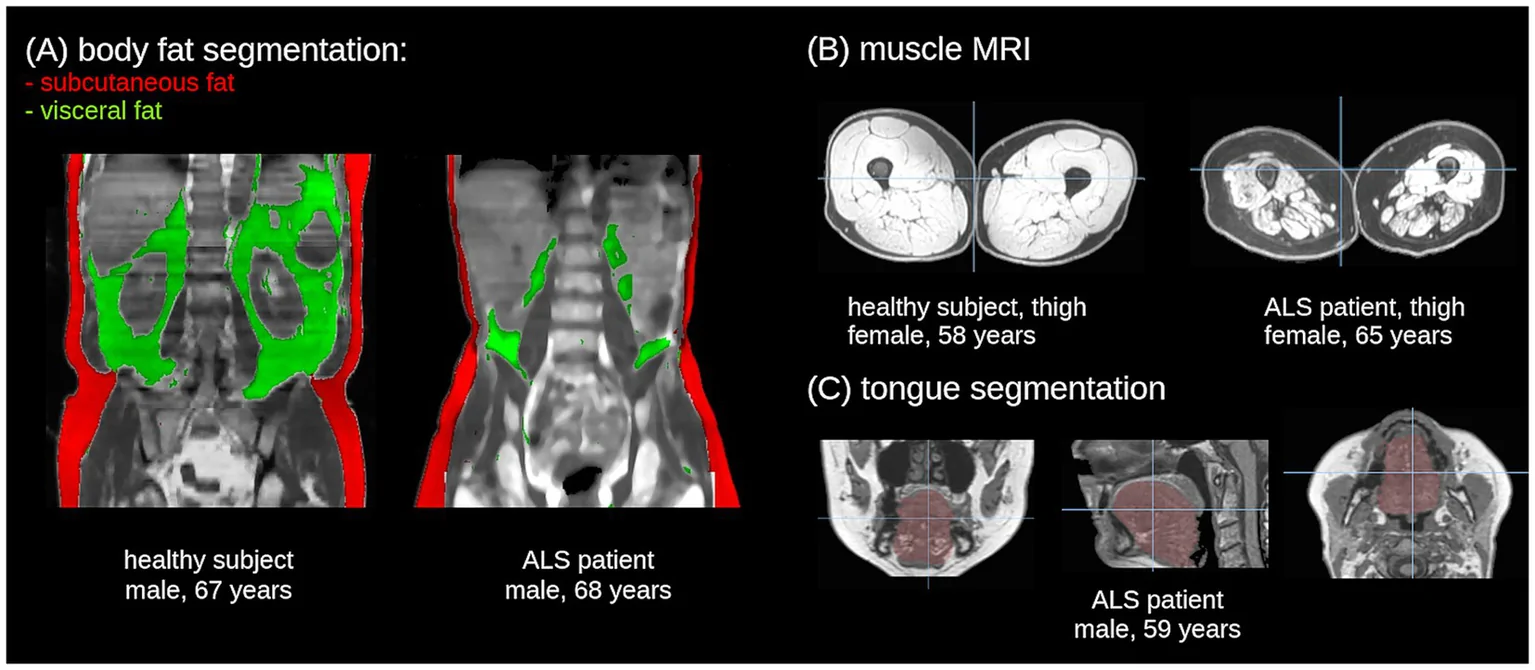
Examples for extra-CNS MRI application in ALS. **(A)** Body fat segmentation (subcutaneous and visceral fat compartments) could be performed from standard T1w. Left image: Healthy subject with body mass index (BMI) of 27, right image, ALS patient with BMI of 19. **(B)** Muscle MRI from a Dixon technique shows differences between healthy subjects and ALS patients with respect to atrophy and signal changes. **(C)** Tongue segmentation could be performed from T1w MRI (MPRAGE).

To date, new MRI techniques have been developed to address the spectrum of extra-CNS applications to ALS. Examples are depicted in Figure 2. However, there is still potential for improvement and the further development of new techniques.

For bodyfat compartment imaging of the abdominal region, an in-plane resolution of 1–3 mm^2^ and an axial slice thickness of 3–5 mm are adequate. Nowadays, imaging protocols (at 1.5 *T* or 3.0 *T*) are capable of acquiring T1- or T2-weighted scans of the abdominal region within a timeframe of up to 10 min (46), although often whole body scans with an acquisition time of less than 30 min were acquired (67, 68), and acquisitions down to 10 min are feasible (Table 1). The application of extended techniques (compared to morphological imaging), such as the Dixon technique (69), enables the separation into pure fat and water images and, thus, also allows for imaging fat/water content for the examination of organs (e.g., the liver (67)).

**Table 1:** Studies of muscle MRI in motor neuron diseases, ordered by reference in alphabetical order.

References	Disease under study	Patients/controls	MRI contrast	Scanning time	Design	Conceptualization
Bryan et al. (73)	ALS	11/8	T1/T2	Not provided	Longitudinal	Volumetric muscle MRI
Cha and Patten (57)	ALS	16/20	T1	Not provided	Cross-sectional	Tongue abnormalities
Dahlqvist et al. (74)	SBMA	40/25	Dixon + T1	Not provided	Cross-sectional	Muscle MRI: fat fraction
De Barros et al. (58)	ALS	43/15	T1 FLAIR	Not provided	Cross-sectional	Tongue volume analysis
Diamanti et al. (37)	ALS	10/9	T1	90 min	Cross-sectional	Muscle MRI: fat infiltration
Diamanti et al. (75)	ALS	43/11	T1	90 min	Cross-sectional	Paraspinal muscle MRI: T1 signal
Diaz et al. (47)	ALS	17/0	Dixon + T2	7 + 10 min	Longitudinal	Muscle MRI: atrophy and fat fraction
El Khalfi et al. (39)	ALS	15/9	DWI + Dixon	34 min	Cross-sectional	Whole body muscle MRI: pattern
Erdal et al. (42)	ALS	22/25	T1	Not provided	Longitudinal	Cervical skeletal muscle index
Foesleitner et al. (48)	MMN/ALS	30/10	T2/DTI/T2 relaxometry	20 min	Cross-sectional	Quantitative MR neurography
Gerevini et al. (76)	ALS	23/12	T2	Not provided	Cross-sectional	Brachial plexus and limb-girdle muscles
Hamano et al. (77)	SBMA/ALS	5/1	T1	Not provided	Cross-sectional	Muscle MRI: atrophy and fatty degeneration
Hensiek et al. (63)	ALS	175/104	T1	10 min	Cross-sectional	Tongue volume
Heskamp et al. (52)	ALS	10/10	DWI/T1	12 min	Longitudinal	Whole-body fasciculations (including tongue)
Jenkins et al. (78)	ALS	4/11	T1	Not provided	Longitudinal	Mmuscle atrophy (including tongue)
Jenkins et al. (79)	MND	29/22	T2	6 min	Longitudinal	Whole-body muscle MRI
Jenkins et al. (38)	MND	29/22	T2/DWI	60 min	Longitudinal	Longitudinal muscle-based biomarker
Klickovic et al. (16)	SBMA/ALS	42/16	Dixon + T1 + STIR	Not provided	Cross-sectional	Skeletal muscle MRI
Kronlage et al. (80)	ALS/MMN	30/15	T2	Not provided	Cross-sectional	Muscle denervation, fascicular swellings
Lee et al. (62)	ALS	2/5	DTI	10 min	Cross-sectional	Tongue impairment
Liang et al. (49)	ALS	47/20	Dixon + T2	8 min	Cross-sectional	Brachial Plexus and limb-girdle muscles
Lindauer et al. (27)	ALS	62/62	T1	27 min	Cross-sectional	Adipose tissue distribution
Rosenbohm et al. (66)	SBMA	39/51	T1	8 min	Cross-sectional	Tongue volume
Shaw et al. (64)	MND	132/103	T1/T2	6 min	Cross-sectional	Tongue musculature MRI signal
Shobe et al. (59)	ALS	1/0	T1	10 min	Cross-sectional	Bright tongue sign
Staff et al. (81)	ALS/MMN	68/0	T1	Not provided	Cross-sectional	Nerve and muscle MRI abnormalities
Vernikouskaya et al. (65)	ALS	39/39	T1	10 min	Cross-sectional	Tongue volume
Vernikouskaya et al. (70)	ALS	74/81	T1	5 min	Cross-sectional	Bodyfat quantification
Vinciguerra et al. (44)	ALS	30/30	T1	10 min	Cross-sectional	Muscle thickness
Whittaker et al. (18)	ALS	4/6	DWI	30 min	Cross-sectional	Motor unit fasciculations
Wimmer et al. (46)	ALS	33/30	Dixon + T1	30 min	Cross-sectional	Whole-body muscle involvement pattern

SBMA, Spinobulbar muscular atrophy; MMN, Multifocal motor neuropathy; DWI, Diffusion weighted imaging; PBP, Progressive bulbar palsy; STIR, Short Tau Inversion Recovery.

For muscle MRI of the limbs, higher resolution T1-weighted images (at least 1 × 1 × 3 mm^3^) are required (37). A list of studies in MND, investigating muscle MRI was summarized in Table 1. Quantitative muscle MRI with Dixon, DTI, and T2-weighted acquisitions was feasible within 10 min in cross-sectional as well as in longitudinal design (82, 83). In summary, adapted acquisition protocols allow for muscle or whole body (or abdominal) MRI within a scan time of much less than 30 min with a resolution sufficient for quantitative analysis; that way sufficient in clinical trials and acceptable for patients with ALS.

Tongue volume alterations initially were a byproduct as an outcome parameter of T1-weighted MRI of the head; thus, tongue movements by the subjects or incomplete recordings of the tongue by inappropriate field-of-view placement could render the recordings unusable for tongue analysis in many studies. Nevertheless, also in retrospective studies, tongue volume analyses from T1-weighted MRI of the head should be feasible in a high percentage of acquisitions. That way, acquisition protocols with less than 15 min (Table 1) are available to allow for quantitative tongue (volume) analysis. Early studies in MND focussed on qualitative analysis or (manual) volumetric analysis. Recent works showed the possibility of automated volumetric analysis for body fat (70) or tongue volumetry (65), paving the way on future automated quantitative muscle analysis. Multi-center studies show the feasibility of inter-scanner harmonisation and, thus, allow for the collection of large subject groups (64).

## Conclusions – research gaps and future directions

Extra-CNS imaging like bodyfat compartment determination and muscle MRI including tongue volumetry can be used for disease monitoring and can help to assess the impact of management strategies on ALS and related conditions. MRI of anatomical regions outside of the CNS shows potential in terms of biomarker development and development of optimal targets for ALS.

Circulating lipids, adipokines, and imaging phenotypes could serve as biomarkers, especially when combined with metabolomics and CNS imaging. Determining whether subcutaneous vs. visceral fat preservation is superior, and identifying the minimal effective fat thresholds, are key questions. Evidence across animal models, large cohort studies, imaging analyses, and mechanistic work has converged on the conclusion that bodyfat – especially subcutaneous adipose tissue – plays a pivotal role in ALS risk, progression, and survival. Fat loss is not merely a symptom but potentially a modifiable therapeutic target (13). Measuring fat stores, integrating fat-targeted nutritional strategies (fat-preservation interventions), and integrating metabolic modulation into personalized ALS care could meaningfully improve clinical management in ALS.

As muscle atrophy progresses, there is an increase in fat mass in the muscle tissue, visible as hyperintense areas in T1-weighted images. Muscle changes may be visible even before clinical symptoms appear. MRI allows the progression of muscle atrophy to be monitored. Compared to invasive procedures such as muscle biopsies, MRI is less invasive. New sequencing techniques and image analysis methods, such as quantitative muscle MRI, could enable an even more precise assessment of muscle changes in ALS in the future (36, 37). In addition, the combination of MRI data with other biomarkers is being researched to improve diagnosis and monitoring. Muscle MRI is a promising tool in the diagnosis and monitoring of ALS. It offers a non-invasive way to visualize muscle changes that can occur at an early stage. Nevertheless, it should currently be seen primarily as complementary to conventional methods such as EMG and clinical examination. With further advances in research, muscle MRI could play an even more important role in ALS diagnostic procedures in the future. As a specific muscle with respect to the core symptom complex of bulbar dysfunction, tongue atrophy correlates with disease progression. Tongue volume could therefore serve as a biomarker to monitor disease progression and improve prognosis. A decrease in tongue volume has been associated with a deterioration in motor function and faster progression of ALS. Some studies suggest that tongue volume may serve as a predictor of survival time. Patients with more severe tongue atrophy tend to have a worse outcome, and tongue volume correlates with the severity of dysphagia and dysarthria, which can have a significant impact on quality of life.

In summary, the research in the area of extra CNS-imaging in ALS is still ongoing and there are no definitive, universally accepted guidelines established for its use as a prognostic marker. There is a need for standardized protocols and reference values. It is thought that extra CNS-imaging in combination with other clinical parameters may help to better predict disease progression. In that context, it has been discussed about the role of imaging in ALS/MND in general (71) that the diagnosis in a given patient is based upon the clinical examination in the first line which is then refined by additional diagnostic tools including electrophysiology, cognitive and behavioral assessment, laboratory diagnostics (including cerebrospinal fluid and perhaps genetic testing), and neuroimaging. The role of cerebral and spinal MRI, i.e., mostly limited to imaging of the CNS yet, has been defined to provide the exclusion of ALS mimics and to establish advanced applications. Here, the implementation of a multiparametric protocol has been proposed for a composite score from different MRI techniques and different anatomical regions which will contribute to the diagnostic pathway to ALS (71). In this line of argument, advanced imaging also of structures outside the CNS like bodyfat and muscles might be integrated into the imaging domain of MND patient assessment, since it can non-invasively guide the clinician in the pattern of clinical involvement of the whole body and its severity. As such, imaging techniques of structures outside the CNS have a differential diagnostic potential and value in pathophysiological understanding (by identifying patterns) and might serve as a biomarker or surrogate marker if assessed quantitatively, especially longitudinally. To this end, however, the clinical application of these multiparametric advanced MRI protocols for the characterization of body tissue outside the CNS in individual patients requires continuous efforts to standardize the MRI acquisition and postprocessing protocols on the basis of multi-center initiatives. In particular, the role of AI-based algorithms remains to be explored in that process (72). With such standardized examinations established and available, whole body MRI (including brain, spine, bodyfat, and muscles) might be conceptualized as an integrative element of personalized medicine in MND with international standards.
